# Supplementary material for: The interactome of intact mitochondria by cross-linking mass spectrometry provides evidence for coexisting respiratory supercomplexes
Source: Mol Cell Proteomics. 2017 Dec 8;17(2):216–32. doi: 10.1074/mcp.RA117.000470 (PMC5795388; doi:10.1074/mcp.RA117.000470)
Supplement: Supplemental Data [file supp_RA117.000470_133922_0_supp_23978_fzffwf.zip › spectra_annotation/mito_DR_spectra_annotation/163-1-3-1-12-1.pdf]

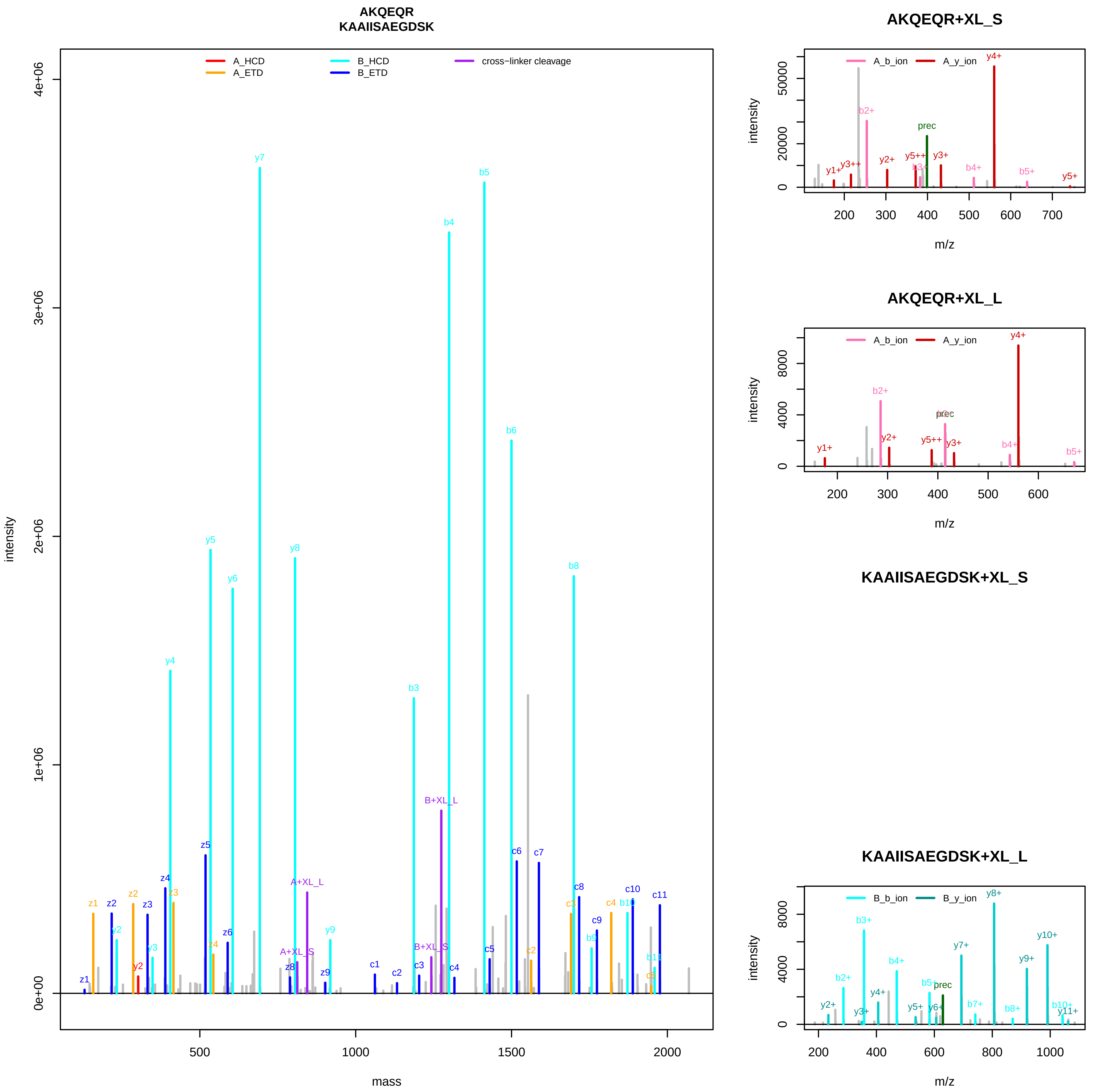
AKQEQR
KAAIISAEGDSK
A_HCD
A_ETD
B_HCD
B_ETD
cross−linker cleavage
intensity
mass
0e+00
1e+06
2e+06
3e+06
4e+06
500
1000
1500
2000
y7
b5
b4
b6
y5
y8
b8
y6
y4
b3
B+XL_L
z5
c6
c7
z4
A+XL_L
c8
c10
c11
z1
z2
z3
z2
z3
c3
c4
b10
y2
y3
y2
z4
z6
y9
c9
b9
A+XL_S
B+XL_S
z8
c1
c2
c3
c4
c5
c2
z9
b11
c5
z1
AKQEQR+XL_S
A_b_ion
A_y_ion
intensity
m/z
0
20000
50000
200
300
400
500
600
700
y4+
b2+
prec
y1+
y3++
y2+
y5++
b3+
y3+
b4+
b5+
y5+
AKQEQR+XL_L
A_b_ion
A_y_ion
intensity
m/z
0
4000
8000
200
300
400
500
600
y4+
b2+
prec
y2+
y1+
y5++
y3+
b4+
b5+
KAAIISAEGDSK+XL_S
KAAIISAEGDSK+XL_L
B_b_ion
B_y_ion
intensity
m/z
0
4000
8000
200
400
600
800
1000
y8+
b3+
y10+
y7+
b2+
y4+
b4+
y9+
b5+
prec
y2+
y3+
y5+
y6+
b7+
b8+
b10+
y11+
